# ELPS helps!

**DOI:** 10.1192/bjo.2021.940

**Published:** 2021-06-18

**Authors:** Rajalakshmi Valaiyapathi, Ksenia Marjanovic-Deverill, Kezia Smith

**Affiliations:** Central North West and West London Mental Health Trust

## Abstract

**Aims:**

This study aimed to identify whether contact with the Ealing Liaison Psychiatry Service (ELPS) improved patients’ mental health using the Clinical Global Impressions (CGI) scale, and to understand the utility of this tool.

**Background:**

CGI is a frequently used outcome measure in psychiatry and also forms part of the RCPsych Framework for Outcome Measures in Liaison Psychiatry (FROM-LP) across the NHS's LP Services. However, there is minimal literature discussing the meaning of the quantitative results of the questionnaire. What would be a cut-off point associated with the provision of good care? It is not possible to draw conclusions about the quality of service and care based on the proportion of the patients who report an improvement on CGI in the absence of a gold standard.

**Method:**

Patients and their ELPS clinicians filled out a CGI questionnaire, rating the patient's mental health condition after contact with the clinician. The 1-7 rated CGI scale indicated the following: 1-3 signified varying degrees of improvement, 4 signified no change and 5-7 signified varying degrees of feeling worse. This study looked at all 205 patients with completed CGI questionnaires who had more than one face-to-face contact with a clinician in 2018 and 2019.

Patient and clinician ratings were compared for concordance and patient notes were reviewed to identify potential reasons for patients with low CGI scores.

Randomised sampling of patients who scored 1 ‘Very much improved’, 2 ‘Much improved’ and 3 ‘Minimally improved’ was conducted to identify differences in number of face-to-face contacts between the groups.

**Result:**

59% of patients reported an improvement, 40% felt that there was no change and 1% (3 patients) indicated feeling worse. Of the latter, 2 patients had been admitted to a mental health unit.

91% of cases showed concordance between patient and clinician ratings.

Randomised sampling identified 9 patients scoring ‘1’, 22 patients scoring ‘2’ and 16 patients scoring ‘3’. The vast majority of patients had only two contacts with ELPS (77%).

**Conclusion:**

ELPS intervention improves patients’ self-reported wellbeing in 59% of patients according to CGI.

There was no correlation between number of face-to-face contacts and the degree to which patients felt better. However, in the absence of a nationally-recognised gold standard, it is not possible to draw conclusions about whether care provided by ELPS is good compared to other services. Data from other centres are required to elucidate what constitutes a gold standard to aspire towards.

